# Metabolic Effects of Cholecystectomy: Gallbladder Ablation Increases Basal Metabolic Rate through G-Protein Coupled Bile Acid Receptor Gpbar1-Dependent Mechanisms in Mice

**DOI:** 10.1371/journal.pone.0118478

**Published:** 2015-03-04

**Authors:** Víctor Cortés, Ludwig Amigo, Silvana Zanlungo, José Galgani, Fermín Robledo, Marco Arrese, Francisco Bozinovic, Flavio Nervi

**Affiliations:** 1 Departamento de Gastroenterología, Pontificia Universidad Católica de Chile, Santiago, Chile; 2 Departamento de Nutrición, Diabetes y Metabolismo, Pontificia Universidad Católica de Chile, Santiago, Chile; 3 Facultad de Medicina, CASEB y Departamento de Ecología, Facultad de Ciencias Biológicas, Pontificia Universidad Católica de Chile, Santiago, Chile; Texas A&M Health Science Center, UNITED STATES

## Abstract

**Background & Aims:**

Bile acids (BAs) regulate energy expenditure by activating G**-**protein Coupled Bile Acid Receptor Gpbar1/TGR5 by cAMP-dependent mechanisms. Cholecystectomy (XGB) increases BAs recirculation rates resulting in increased tissue exposure to BAs during the light phase of the diurnal cycle in mice. We aimed to determine: 1) the effects of XGB on basal metabolic rate (BMR) and 2) the roles of TGR5 on XGB-dependent changes in BMR.

**Methods:**

BMR was determined by indirect calorimetry in wild type and Tgr5 deficient (Tgr5^-/-^) male mice. Bile flow and BAs secretion rates were measured by surgical diversion of biliary duct. Biliary BAs and cholesterol were quantified by enzymatic methods. BAs serum concentration and specific composition was determined by liquid chromatography/tandem mass spectrometry. Gene expression was determined by qPCR analysis.

**Results:**

XGB increased biliary BAs and cholesterol secretion rates, and elevated serum BAs concentration in wild type and Tgr5^-/-^ mice during the light phase of the diurnal cycle. BMR was ~25% higher in cholecystectomized wild type mice (p <0.02), whereas no changes were detected in cholecystectomized Tgr5^-/-^ mice compared to wild-type animals.

**Conclusion:**

XGB increases BMR by TGR5-dependent mechanisms in mice.

## Introduction

Cholecystectomy (XGB) is one of the most frequent surgical procedures worldwide and is recognized as safe procedure with no impact on metabolic regulation whatsoever. Emerging evidence, however, associates XGB with excessive risk for metabolic syndrome and associated complications in humans, including diabetes type 2, arteriosclerotic vascular diseases and non—alcoholic fatty liver disease (NAFLD) [1– 6]. We have reported that XGB results in elevated serum and liver triglyceride content, and VLDL production in mice, although the mechanisms for this association still remain elusive [7]

Gallbladder (GB) controls whole body bile acids (BAs) kinetics. In fact, XGB significantly shortens BAs recirculation time in both mice [7] and humans [8–10]. This results in elevated exposure of enterohepatic and peripheral tissues to BAs during the fasting periods of the diurnal cycle. Since BAs have recognized regulatory roles in energy, glucose and lipid metabolism (reviewed in [11–16]), we have hypothesized that XGB may determine whole body energy homeostasis deregulation. Metabolic actions of BAs are mediated by nuclear and cell surface receptors. Among them, the nuclear receptor FXR and the membrane G protein-coupled receptor TGR5 (Gpbar-1, G-protein coupled bile acid receptor), are the best studied and possibly the more biologically significant since they are the only ones activated by BAs at physiological concentrations [11, 12].

FXR, also termed as Nr1h4, belongs to the heterodimeric nuclear receptor superfamily and transcriptionally regulates several enzymes and transporters involved in BAs, fatty acids, triglycerides, cholesterol and carbohydrate metabolism [11–13]. FXR is mainly expressed in the liver, intestine, adipose tissue, pancreas and kidney. After its activation by BAs, it forms heterodimers with Retinoid X Receptor (RXR) and binds to FXR response elements (FXRE) in the promoter region of its target genes. In hepatocytes, FXR regulates *Cyp7a1*expression, the gene encoding for the BAs biosynthesis rate-limiting enzyme. In ileal enterocytes, BAs activated FXR enhances Fibroblast Growth Factor 15 (FGF15, human ortholog FGF19) production and release. FGR15/19 reaches the liver and represses *Cyp7a1* gene transcription through a FGF Receptor 4 and β Klotho dependent mechanism [11]. Besides BAs homeostasis, FXR is involved in insulin sensitivity and glucose and lipid regulation. It, therefore, may have important roles in metabolic syndrome and associated diseases [12, 13]. Recently, a direct role for FXR in energy homeostasis was described [17]. In fact, *Fxr* ^-/-^ mice are notoriously resistant to age-related obesity and have increased energy expenditure [17].

TGR5 is ubiquitously expressed in human and rodent tissues, including brown adipose tissue, nervous system, muscle and gastrointestinal tract [18–20]. In particular, TGR5 gastrointestinal immunoreactivity includes GB mucosal cells, cholangiocytes, Kuppfer cells and small intestine enterocytes. It also has a prominent expression in the enteric nervous system (Meissner’s submucosal and Auerbach’s myenteric neuronal plexuses), implying that this receptor may have roles in the regulation of intestinal secretion, absorption, motility and blood flow [19]. TGR5 has important metabolic effects. TGR5 gene deletion favors obesity and excessive hepatic triglyceride accumulation in mice [18]. Conversely, TGR5 activation increases energy expenditure in BAT and prevents fat accumulation in the liver [21]. In the small intestine, TGR5 positively regulates glucagon-like polypeptide-1 (GLP1) secretion by L enteroendocrine cells and thus favorably modulate insulin sensitivity and glucose metabolism [22].

Whole body energy expenditure is composed of basal metabolism, adaptive thermogenesis and muscular activity, and is influenced by a number of interrelated nervous and hormonal factors modulated by extrinsic variables. Basal energy expenditure or basal metabolic rate (BMR) [23, 24] is mainly determined by mitochondrial ATP production for synthesis and transport processes and represents the minimal energy cost of living [25]. It remains unknown whether physiological circadian fluctuations of circulating BAs or pathological changes in BAs recirculation kinetics significantly influence BMR. Thus, the main aim of the present study was to address the effects of XGB on BMR in mice. We explored whether western type diet (WD) induced obesity modify BMR response to XGB and what was the role of TGR5 on GB-dependent BMR regulation.

## Materials and Methods

### Animals and diets

C57BL/6 mice were obtained from The Jackson Laboratories (Bar Harbor, Main, USA). Tgr5^-/-^ mice were kindly provided by Prof. Johan Auwerx (Laboratory of Integrative and Systems Physiology, School of Life Sciences, École Polytechnique Fédérale de Lausanne, Switzerland). Mice were housed in colony cages at 25°C in a ventilated room with a 12- hour light/dark cycle and unrestricted access to food and water. Animals were fed either a chow diet (Prolab, PMI Nutritional International, Brentwood, MO, USA), containing 26% protein, 14% fat, and 60% carbohydrates (expressed as % total calories), and total 4.1 kcal/g, or a Western-like diet (WD) made of chow diet supplemented with 20% (wt/wt) milk fat, providing 17% protein, 44% fat, and 39% carbohydrates (expressed as % total calories), and total 5.3 kcal/g. Adult 5 to 6 weeks old male C57BL/6 and *Tgr5* ^-/-^ mice, weighing 19–23 g were subjected to XGB or sham operation. All the animal procedures were approved by Pontificia Universidad Católica de Chile School of Medicine animal studies ethics committee, in agreement with the Public Health Service (PHS) Policy on Humane Care and Use of Laboratory Animals recommended by the Institute for Laboratory Animal Research (ILAR) Guide for Care and Use of Laboratory Animal.

### Cholecystectomy

XGB and sham operation were performed as described [7]. Briefly, overnight fasted animals were anesthetized with isoflurane, abdominal skin was wiped with 70% alcohol and the abdominal cavity was accessed through a small medial laparotomy. The cystic duct was ligated and the GB was emptied with a 24-gauge needle syringe. Afterwards, the gallbladder was gently removed by surgical dissection and the abdominal wall was closed by manual suture with catgut. Abdominal skin was repaired with a silk suture that was removed 7 days after the procedure. Control mice were subjected to a sham-operation [7]. Postoperative analgesia was provided with ibuprofen for 7 days. Surgery was well tolerated as indicated by the normal body weight gain along the observational period.

### Bile, blood and tissue sampling

Specimens were obtained at the middle of the light phase or at the middle of the dark phase of diurnal cycle, as previously described [7]. Prolonged fasting was avoided because of the rapid fuel conversion in mice [26, 27].

### Plasma bile acids

BAs specific composition was measured in 25 μl of pooled plasma. Equal volume aliquots of individual plasma were combined to generate pools of each experimental group and centrifuged at 15,900 *g*. The resulting supernatant was saved, evaporated under vacuum at 40°C and reconstituted in 100 μl of 50% methanol. BAs species were determined by liquid chromatography tandem mass spectrometry (LC-MS/MS) as described [28]. Known amounts of D4-cholate, D4-chenodeoxycholate, D4-glycocholate, D4-taurocholate, D4-glycochenodeoxycholate and D4-taurochenodeoxycholate were added as internal controls.

### Basal metabolic rate

Basal metabolic rate (BMR) was estimated by the oxygen consumption (VO_2_) in thermoneutrality (30.0 0.5°C) and under post-absorptive conditions. Individual animals were housed in plexiglass metabolic chambers (1000 mL) and VO_2_ was measured with a Datacan V open-flow respirometry system (Sable Systems, Henderson, NV, USA) in the middle of the light phase of the diurnal cycle (corresponding to period of lowest spontaneous activity) [29, 30]. Metabolic chamber were ventilated with dried CO_2_-free air at a constant rate of 800 mL/min (Sierra Instruments, Monterey, CA, USA). Body mass was measured prior to metabolic measurements using an electronic balance (± 0.1 g). BMR was estimated by the lowest VO_2_ in a period of 3 minutes. Each individual BMR determination procedure lasted at least for 2 hours.

### Brown adipose tissue, liver and skeletal muscle tissue palmitate oxidation

Muscle, liver and interscapular BAT (~80 mg) was homogenized while maintained on ice in a glass tube containing modified sucrose-EDTA medium. Palmitate solution was fresh prepared on the day of testing by combining 10 μL of 1 μCi/ml [1-^14^C]-palmitate (Perkin Elmer. Waltham, MA, USA]) and 17–200 μL of 20 μM of unlabeled palmitate. Palmitate was complexed to fatty acid-free BSA (MP Biomedicals) for 30 min (37°C) at a constant palmitate-to-BSA molar ratio (3/1). Palmitate oxidation was determined in triplicate at 45, 90, 180 and 520 μM palmitate by measuring production of ^14^C-labeled acid-soluble metabolites (ASM) and ^14^CO_2_ from the carboxyl carbon. Reactions were initiated by adding 310 μl of the incubation buffer (pH 7.4) to 80 μl of homogenate. Reaction buffer contained 102 mM sucrose, 80 mM potassium chloride, 10 mM potassium phosphate, 8 mM Tris·HCl, 2 mM ATP, 1 mM magnesium chloride hexahydrate, 1 mM L-carnitine, 1 mM dithiothreitol, 0.2 mM EDTA, 0.1 mM malate, 0.1 mM NAD^+^, 0.05 mM CoA and 0.5% fatty acid-free BSA. After incubation (60 min, 37°C), reactions were terminated by adding 40 μl of 70% perchloric acid, and the produced CO_2_ was trapped in 200 μl of 1 M NaOH. The acidified medium was stored overnight (4°C), and then ASM assayed in supernatants. Radioactivity of CO_2_ and ASM were determined by liquid scintillation counting and normalized for muscle-wet weight [31].

### Blood and bile biochemistry

Blood glucose was determined with ACCU-CHECK glucometer (Roche diagnostics, Mannheim, Germany). Serum alanine aminotransferase (ALA) was measured by ALAT (Kovalent, Rio de Janeiro, Brazil) following manufacturer’s instructions. Serum insulin and thyroxin were determined by ELISA (Crystal Chem., Downers Grove, IL, USA, and Endocrine Technologies, Newark, CA, USA, respectively). Brown adipose tissue cyclic adenosine monophosphate concentration (cAMP) was quantified by ELISA (Cell Biolabs, San Diego, CA, USA). Total BAs and cholesterol in the bile were measured as previously described [32].

### Total RNA extraction and real—time quantitative PCR analysis

RNA was isolated from the liver and interscapular brown adipose tissue with Trizol reagent following manufacturer’s instructions (Life Technologies, Carlsbad, CA, USA). 2 μg of total RNA were reverse transcribed using Super Script First-Strand Synthesis System for RT-PCR (Invitrogen, Carlsbad, CA, USA) and random hexamers. Mx3000P Real-Time PCR System (Stratagene, La Jolla, CA, USA) was used for quantitative PCR using Platinum Quantitative PCR Super Mix-UDG (Invitrogen) and 25 ng of cDNA as template. Primers sequences for uncoupling protein 1 (UCP1) were: (fw) GAGGTGTGGCAGTGTTCATTG and (rev), GGCTTGCATTCTGACCTTCA; for UCP2 (fw) GCTTCTGCACCACCGTCAT and (rev), GCCCAAGGCAGAGTTCATGT; and for UCP3 (fw) GCTTCTGCACCACCGTCAT and (rev), GGCCCTCTTCAGTTGCTCAT. Thermal cycling conditions were 95°C for 10 minutes followed by 40 cycles at 93°C for 15 seconds, and 60°C for 1 minute. Reactions were performed in duplicate. Relative quantification of gene expression was performed using the comparative threshold method as described by Applied Biosystems (Foster City, CA, USA) using cylophilin as housekeeping reference gene.

### Statistics

Data are presented as means ± SE. Comparison between samples and statistical analysis were performed using Prism 5.0 (Graphpad software La Jolla, CA, USA). ANOVA and two-tailed unpaired Student’s *t*-test were used to compare functional sets of data. Significant differences were considered at p value < 0.05.

## Results

### General metabolic effects of XGB in mice

**Fig 1:**
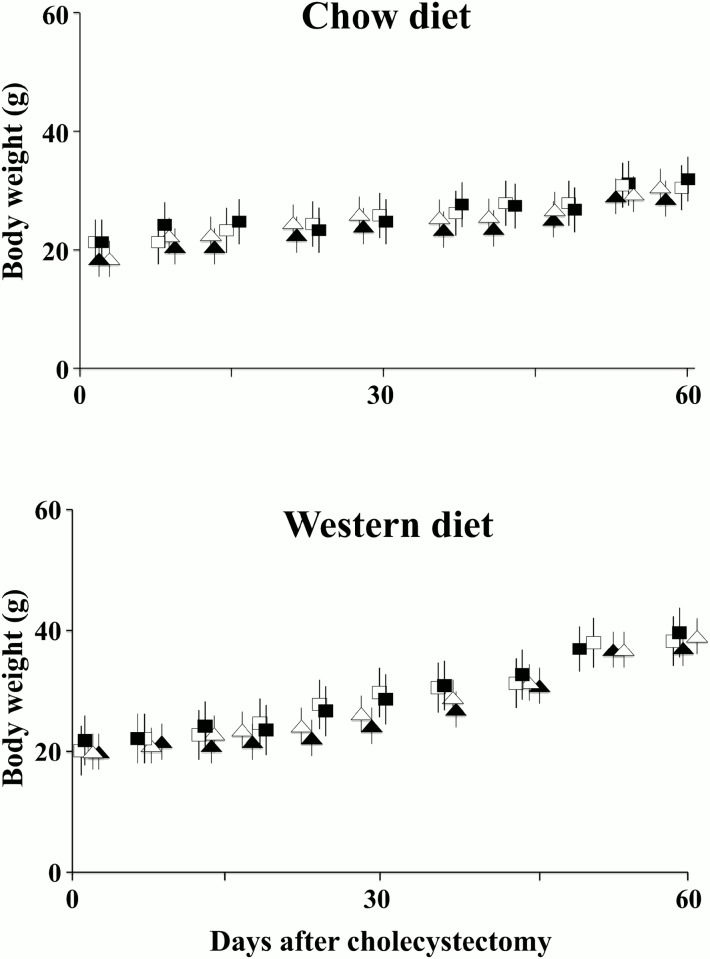
Body weights after XGB in wild type and *Tgr5* ^-/-^ mice fed chow or WD. This figure presents 12 wild type and 6 *Tgr5* ^*-/-*^sham-operated mice or subjected to XGB. Initial weight of wild type and 6 *Tgr5* ^*-/-*^ mice were 19 to 23 g. Values represent the mean ± S.E.M. Final weights at the specific experiments 37–40 days after surgery were significantly increased by 53% to 65% in all groups of mice fed the WD compared to groups of mice fed the normal chow (p< 0.02). These differences were maintained throughout the observational period of 60 days. XGB had no effects on body weight changes. Values represent the mean ± S.E.M. Squares represent wild—type and triangles represent *Tgr5* ^*-/-*^ mice. White figures and black figures represent sham—operated and mice subjected to XGB, respectively.

In order to evaluate the actions of the gall bladder (GB) on energy homeostasis, male mice were subjected to XGB and fed either chow or western type diet (WD). Mice subjected to sham operation and fed same diets were used as controls. All animals remained healthy as indicated by equivalent body weight gain rates in each group. Fig. 1 shows body weight changes in animals fed either control chow (panel A) or WD (panel B) followed for 60 days on the diets. As expected, animals fed the WD gained significantly more weight compared to the animals fed normal chow diet. Wild type and *Tgr5* ^-/-^ mice evolved with similar body weights increase as a function of time. No differences were observed in animals with XGB compared to sham operated mice in both groups of animals.

All specific experiments were performed after 37 to 42 days on the specific diets. Diet intake was monitored for 4 days in individual cages in a series of 5 to 8 mice in each group after 37 days on the diets. Normal chow intake was 4.7 ± 0.6 g/day and 4.9 ± 0.7 g/day in sham operated wild type and *Tgr5* ^-/-^ mice, respectively (p, NS). No differences were observed in animals with XGB compared to sham operated mice in both groups of animals. Daily WD intake was 4.9 ± 0.5 g/day and 5.0 ± 0.8 in wild—type and *Tgr5* ^-/-^ mice, respectively (p, NS). Both groups of animals subjected to XGB had similar daily food intakes (results not shown).

Wild type and *Tgr5* ^-/-^ mice fed the WD approximately doubled body weight increase compared to animals fed the chow diet. Wild type and *Tgr5* ^-/-^ mice fed the chow diet for 37 days increased body weight 3.0 ± 0.2 g and 3.3± 0.4 g, respectively. Mice fed the WD increased body weight 6.7± 0.8 g (wild type mice) and 6.5 ± 0.8 g (*Tgr5* ^-/-^), (p<0.02 compared to mice fed the chow diet). Importantly, XGB did not influence body weight gain in wild type or *Tgr5* ^-/-^ mice (results not shown). Blood glucose, insulin and thyroxine concentrations also remained unchanged in all groups of mice (Table 1).

**Table 1:** Serum parameters in control and cholecystectomized mice.

	Wild type				*Tgr5* ^-/-^			
Diet	Chow		WD		Chow		HFD	
Surgery	Sham	XGB	Sham	XGB	Sham	XGB	Sham	XGB
ALT (U/L)	34.8 ± 5.6	34.7 ± 4.7	35.3 ± 5.1	30.0 ± 4.7	34.9 ± 5.0	31.7 ± 5.9	35.1 ± 5.5	33.8 ± 4.2
Glucose (mg/dl)	212 ± 18	252 ± 31	219 ± 24	277± 28	233 ± 32	257 ± 38	245 ± 16	262 ± 24
Insulin (ng/ml)	1.3 ± 0.3	1.7 ± 0.4	1.2 ± 0.3	1.6 ± 0.4	1.2 ± 0.3	1.5 ± 0.4	1.4 ± 0.3	1.2 ± 0.4
Thyroxine (μg/dl)	4.7 ± 0.5	4.3 ± 0.4	4.5 ± 0.5	4.0 ± 0.4	4.3 ± 0.5	4.6 ± 0.7	4.8 ± 0.5	4.2 ± 0.4

Values correspond to the mean ± SE. Each experimental group had 6 to 8 individual mice. Blood specimens were obtained at the middle of the light phase of the diurnal cycle. WD, western diet. XGB, mice with cholecystectomy.

### Cholecystectomy increases biliary BAs and cholesterol secretion rates, but serum BAs levels are increased exclusively in the light phase of the diurnal cycle

**Fig 2:**
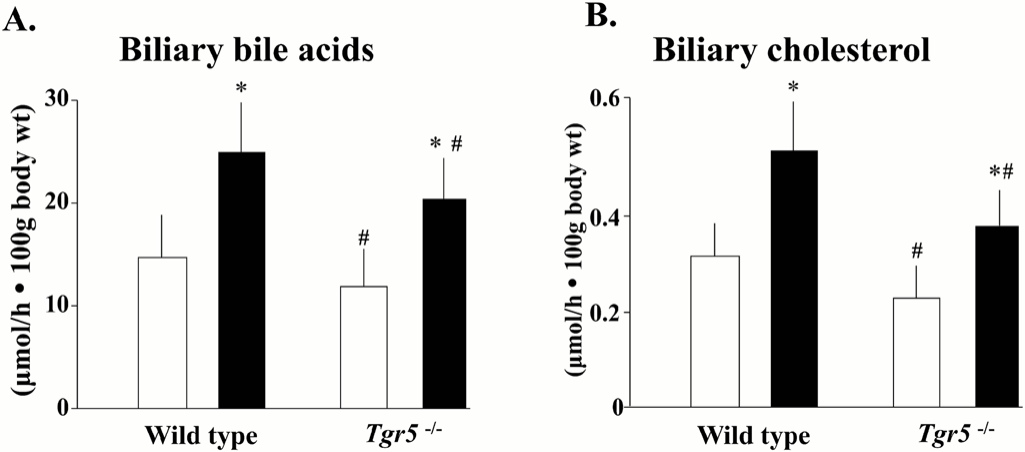
Effect of XGB on biliary BAs and cholesterol secretion rates in wild-type and *Tgr5* ^-/-^ mice. Panel A represents biliary BAs secretion rates and panel B represents biliary cholesterol secretion rates measured 40 to 45 days after XGB or sham operation. Bile fistulas were established and bile flow was measured at the middle of the light phase of the diurnal cycle. White bars represent sham—operated mice and black bars represent mice subjected to XGB. There were 5 to 8 animals in each group. Values represent the mean ± S.E.M. *p<0.02; ^#^p <0.05 compared with the wild type animals.

Biliary BAs and cholesterol secretion rates were measured in the middle of the light phase of the diurnal cycle. As shown in Fig. 2, biliary BAs and cholesterol secretion rates were lower in *Tgr5* ^-/-^ mice in comparison with wild type animals (p < 0.05) and cholecystectomy elevated biliary BAs and cholesterol secretion rates in both wild type and *Tgr5* ^-/-^ mice. Reportedly, *Tgr5* ^-/-^ mice have smaller BAs pool size than wild type mice [18], thus the lower biliary BAs in *Tgr5* ^-/-^ mice can be the mere result of this smaller pool size. The decrease of biliary cholesterol output in *Tgr5* ^-/-^ mice is also the consequence of the decrease of BAs output, since cholesterol secretion is tightly coupled to BAs secretion rate [32].

**Fig 3:**
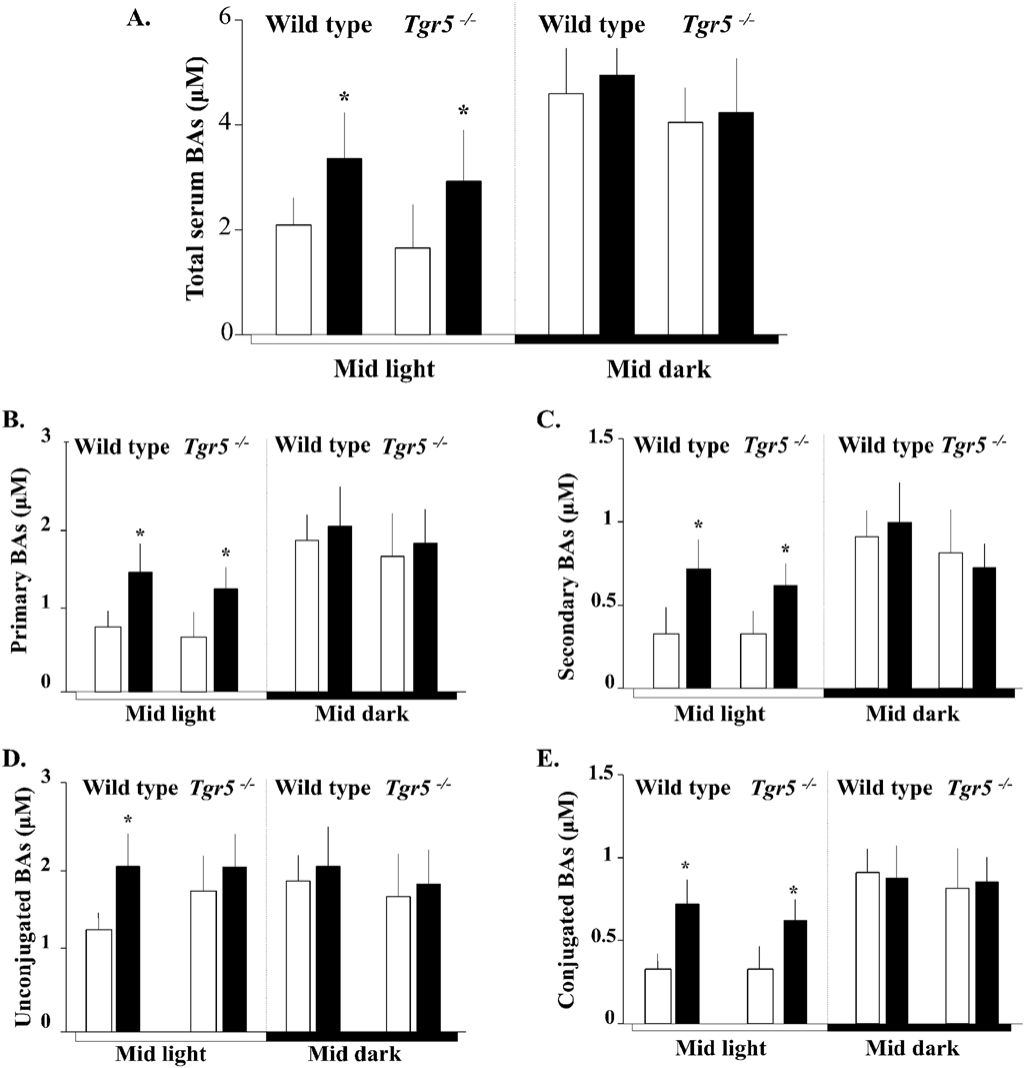
Effect of XGB on serum BAs concentration in wild-type and *Tgr5* ^-/-^ mice fed a chow diet. Blood specimens were obtained at the middle of the light and dark phases of the diurnal cycle to 5–6 wild—type and 4–5 *Tgr5* ^-/-^ mice in each group of animals. White bars represent sham—operated mice and black bars represent mice subjected to XGB. Panel A shows total serum BAs concentration. Primary and secondary serum BAs concentrations are shown in panels B and C, respectively. Conjugated and unconjugated serum BAs levels are shown in panels D and E, respectively. Values represent the mean ± S.E.M. *p<0.01.

GB removal determined a significant rise in total circulating BA concentration (~ 30%) in wild type and *Tgr5* ^-/-^ mice, as compared with sham-operated animals, but this change was exclusively observed in the light phase of the diurnal cycle (Fig. 3A). Similar results were obtained with primary and secondary BAs (Fig. 3B and 3C), as well as with unconjugated and conjugated BAs (Fig. 3D and 3E). Relative composition of different species of BAs is shown in Table 2. Cholecystectomy did not modified serum BAs composition in mice. No differences were detected either between wild type and *Tgr5* ^-/-^ mice (Table 2).

**Table 2:** Serum BAs composition in wild type and *Tgr5* ^-/-^ mice subjected to sham operation or cholecystectomy.

Genotype	Wild type		*Tgr5* ^-/-^	
Surgery	Sham	XGB	Sham	XGB
Cholic (%)	25 ± 3.1	28 ± 3.6	26 ± 4.8	27 ± 4.6
Chenodeoxycholic (%)	10 ± 1.8	5 ± 0.7	10 ± 1.6	5 ± 1.3
Deoxycholic (%)	14 ± 2.3	16 ± 1.7	14 ± 2.0	16 ± 2.1
Ursodeoxycholic(%)	3 ± 0.5	4 ± 0.5	5 ± 1.1	5 ± 1.4
α Muricholic (%)	20 ± 2.4	17 ± 1.8	16 ± 3.1	15 ± 2.6
β Muricholic (%)	18 ± 2.0	18 ± 2.6	19 ± 3.9	23 ± 3.7
ω Muricholic (%)	10 ± 1.2	12 ± 1.2	10 ± 2.3	9 ± 2.5

Values correspond to the mean ± SE of 5–7 wild type mice and 3–4 *Tgr5* ^*-/-*^ mice. Concentration of serum lithocholic acid was < 1% (< 0.05 μM) in all groups of animals. XGB, cholecystectomy.

### Cholecystectomy increases BMR by TGR5 dependent mechanisms in mice

**Fig 4:**
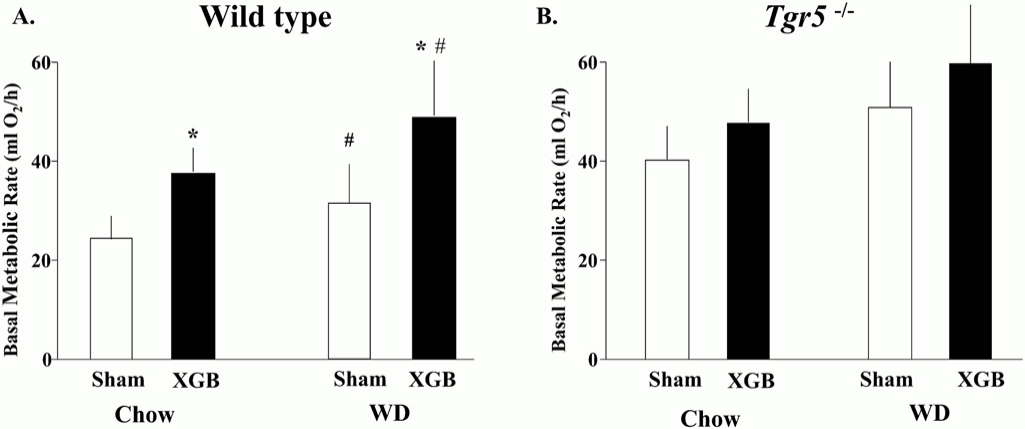
Effect of XGB on basal metabolic rate in wild type and *Tgr5* ^-/-^ mice fed chow or WD diet. VO2 was measured during the dark and light phases of the diurnal cycle in thermoneutral conditions (18, 25). Panel A shows animals fed a control chow diet and panel B represents animals fed the WD. There were 5 to 6 mice per group. White bars represent sham—operated mice and black bars represent mice subjected to XGB. The experiment was independently repeated three times with equivalent results. Values represent the mean ± S.E.M. * p < 0.02 compared with the XGB mice; ^#^ p < 0.05 compared with the wild type animals (two-way ANOVA).

As shown in Fig. 4A, cholecystectomy significantly increased BMR (~25%) in wild type mice fed either chow or western type diet (WD). In contrast, GB removal did not changed BMR in *Tgr5* ^-/-^ mice (Fig. 4B). Unexpectedly, *Tgr5* ^-/-^ mice had elevated BMR in comparison with wild type mice fed both types of diet (p < 0.02), suggesting that TGR5 plays important roles in BMR regulation in mice.

### Cholecystectomy does not change BAT, liver or skeletal muscle fatty oxidation rates or UCP-1 mRNA levels in brown adipose tissue, and UCP-2, UCP-3 in muscle

**Fig 5:**
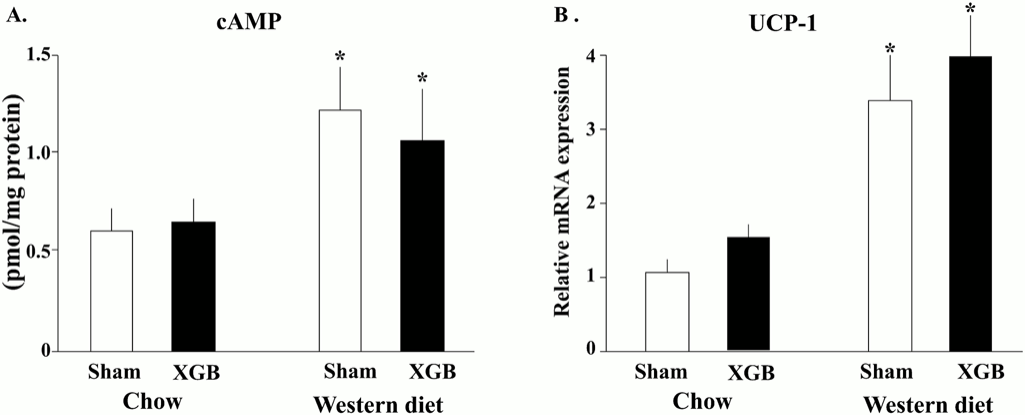
Effect of XGB on BAT cAMP concentration and mRNA expression levels of UCP-1 in wild type and *Tgr5* ^-/-^ mice fed chow or WD diet. Forty to 45 days after XGB animals were sacrificed and tissue samples harvested as described in “Methods”. There were 4 to 8 animals per group. White bars represent sham—operated mice and black bars represent mice subjected to XGB. Values represent the mean ± S.E.M. *p<0.001 compared to animals fed WD.

To explore mechanisms underlying BMR rise after GB ablation, palmitate oxidation rates were measured *ex vivo* in BAT, liver and skeletal muscle tissue homogenates. As shown in Table 3, no differences were found between mice subjected to cholecystectomy and sham-operated animals. As BAT oxidative capacity relies mostly on uncoupled mitochondrial respiration, mRNA levels of uncoupling protein 1 (UCP-1) and cAMP concentration in BAT were determined. Cholecystectomy did not change UCP-1 mRNA levels and cAMP concentration in BAT (Fig. 5). Skeletal muscle UCP-2 and UCP-3 mRNA levels also remained unchanged (results not shown).

**Table 3:** *Ex vivo* palmitate oxidation in brown adipose (BAT), liver and skeletal muscle tissue in cholecystectomized mice.

	Sham	XGB
**Liver**	215 ± 23	176 ± 24
**Muscle**	87 ± 15	112 ± 37
BAT*	369 ± 212	405 ± 28

Values correspond to the mean ± SD (nmol/mg of wet tissue/h) of 3 animals per group. Tissue samples were harvested and processed as described in “Methods”. Data correspond to total plamitate oxidation rate. No significant differences were found between groups.

* BAT, brown adipose tissue.

## Discussion

XGB is recognized as a safe procedure with no deleterious effects on health. However, a growing body of evidence contradicts this common wisdom, in fact showing that GB removal associates with increased risk for various metabolic disarrangements, including insulin resistance and fatty liver [1–3]. Concordantly, we previously found that XGB associates with elevated levels of triglycerides in the liver of mice [7], suggesting previously unrecognized roles for GB in metabolic regulation. Furthermore, since GB regulates BAs recirculation kinetics, it is plausible that GB also contribute to BMR regulation. This contention is supported by recently described roles of BAs in energy balance, body weight, and serum glucose and insulin sensitivity [12–16].

Herein we report that GB ablation determines increased BMR, in association with increased biliary BAs and cholesterol secretion rates and elevated serum BAs levels during the light phase of the diurnal. The finding that XGB does not significantly modify BMR in *Tgr5* ^-/-^ mice indicates that TGR5 is required for GB-dependent energy regulation. We did not find differences in UCP-1 and UCP-2/UCP-3 mRNA levels in BAT and muscle respectively. More importantly, we failed to find differences in fatty acid oxidation rates in BAT, skeletal muscle and liver. No changes were noted in circulating thyroid hormone or BAT cAMP content either. However, since *ex vivo* fatty oxidation studies lack the entire regulatory network that modulate BMR *in vivo*, is not possible to exclude regulatory actions of GB in substrates oxidation in BAT, liver or skeletal muscle as the cause elevated BMR after XGB. TGR5 is expressed in the epithelium of the GB in both mice and humans and its activation increases cell cAMP content [33] and regulates GB filling [34]. It is possible that XGB results in the suppression of a TGR5 dependent metabolic signal emanated from the GB with systemic actions on energy homeostasis.

We propose that increases of BAs recirculation kinetics, resulting from GB ablation, determine increased activation of TGR5 and, thus, higher BMR. In fact, XGB increases enterohepatic recirculation of BAs in the light phase of the diurnal cycle in mice [7]. Noteworthy, the elevation in BMR induced by XGB did not associate with changes in body weight nor prevented diet induced obesity. Concordantly, no changes in serum glucose and insulin levels were noted. It is possible that subtle changes in food intake, or total energy expenditure counterbalance XGB-induced BMR raise, determining a final steady global energy balance. Longer observation periods might be required to unveil the effects of XGB on body weight regulation.

XGB does not change BAs pool size or synthesis rates, but significantly speeds up BAs secretion and recirculation rates [1–3]. We propose that the increased metabolic energy expended to maintain accelerated BAs kinetic, may contribute to elevated BMR after XGB. Secretion of BAs towards the bile and ileal BAs reabsorption are both energy-consuming processes (reviewed in [35–37]). BAs are transported across the sinusoidal membrane by the Na^+^ taurocholate co-transporting polypeptide with the assistance of members of the organic anion transporting polypeptide family and then secreted by ATP-consuming mechanisms across the canalicular membrane by BSEP/ABCB11 [35, 36]. Biliary secretion of cholesterol and phospholipids are also ATP-consuming processes mediated by transporters ABCG5/8 and ABCB4, respectively [38–40]. In the lumen of the small intestine, BAs are reabsorbed by ATP- dependent apical sodium-dependent BAs transporter (ASBT). BAs are then transported across the basolateral membrane of enterocytes and effluxed to the portal circulation by heteromeric organic solute transporter α/β (OSTα-OSTβ) [41, 42]. All these energy—consuming processes are maximally active during the feeding periods (dark phase in rodents) when the GB remains contracted and the BAs pool recirculates faster through the enterohepatic circulation. After XGB in mice, BAs enterohepatic circulation is also high during the fasting period (light phase of the diurnal cycle) [7]. This phenomenon supports the hypothesis that increased energy—dependent transport processes in the enterohepatic circulation after XGB determines elevated BMR during the diurnal cycle in mice.

A number of recent studies support the hypothesis that BAs have important roles in metabolic control, acting as through FXR, FGF15/19 and TGR5 dependent signaling systems [11–14]. Watanabe et al. showed that the administration of the bile acid bind resin colestimide significantly increased total energy expenditure in mice and prevented high fact diet induced obesity, insulin resistance and hepatic steatosis [43]. Interestingly, these authors reported only a minor decrease in the BAs pool size and no changes in the total circulating BAs concentration as a result of colestimide administration. Notably, dietary supplementation of cholic acid had equivalent actions on energy homeostasis albeit completely opposite effects on BAs pool size, plasma BAs concentration and hepatic gene expression profile than colestimide [43]. Watanabe et al suggest that this paradox might be solved by the equivalent rise in taurocholic acid that both colestimide and cholic acid induce. Based on our findings we propose that interruption of normal circadian fluctuations in BAs recirculation might alternatively explain the energy expenditure changes induced by colestimde and cholic acid. Complementing results were provided by pharmacological activation of FXR. Administration of the synthetic FXR agonist GW4064 decreased energy expenditure and accentuated high fat diet induced obesity and glucose intolerance along with decreased BAs pool in mice [44], further supporting a role for BAs in whole body energy regulation.

In the present study we observed that *Tgr5* ^-/-^ mice have increased BMR in comparison with wild-type animals (both sham operated or subjected to XGB). The mechanisms for this difference remain unknown. TGR5 increases energy expenditure by promoting BAT uncoupled mitochondrial oxidation and its pharmacological activation prevents diet-induced obesity [20]. However, since TGR5 is widely expressed across the gastrointestinal tract and its functional significance has mostly been assessed by cell culture studies, with scarce information on its functional roles in the whole animal [12–14]. Future tissue specific *Tgr5* deletion studies will be required to unclose the mechanism for its actions on BMR.

Importantly, evidence for a role of BAs and its signaling systems in human energy expenditure regulation is still lacking. The administration of BAs binding resin colesevelam modestly improved glycemic control in a small cohort of type 2 diabetes patients but although there was a significant increased in BAs synthesis rate, no correlation was found between markers of insulin resistance/glucose metabolism and BA metabolism in these patients [45]. In another study by the same gorup, a BAs sequestrant failed to modify energy expenditure in diabetic patients as well as in non-diabetic control subjects [46].

In summary, we have reported that surgical ablation of GB results in increased BMR in mice, suggesting that, besides its role in lipid digestion and absorption, the GB plays a critical role for systemic energy balance. This function is likely related to its regulatory roles on BAs enterohepatic recirculation kinetics and, thus, the interaction of BAs on TGR5 receptor. These results suggest that XGB, one of the most frequently performed surgical procedures worldwide, may not be as innocuous as previously believed and may have important metabolic implications for humans.
